# The Evolution of the Isolation Hospital
*The previous article appeared on November 22.


**Published:** 1913-12-06

**Authors:** H. Franklin Parsons

**Affiliations:** late Assistant M.O. L.G.B.


### THE EVOLUTION OF THE ISOLATION HOSPITAL.*

By the late H. FRANKLIN PARSONS, M.D., D.P.H., late Assistant M.O. L.G.B.

Coming now to the Metropolis the development of the isolation-hospital system has followed different lines, having fallen into the hands of a Poor-Law body, the Metropolitan Asylums Board, instead of into those of the sanitary authorities or the County Council.

It has been already mentioned that in former times persons suffering from infectious diseases who were without proper lodging and accommodation at their own homes, were commonly treated in the workhouse infirmaries, where they were a danger to the other patients, and where the lodging and nursing provided were often of a very poor order. It was at one time the aim of the Poor-Law Board to promote the erection of suitable fever wards in detached blocks, but it was found later on that the provision of such wards by no means met the needs of the community for means of isolation of infectious diseases. Admission to such buildings was by law restricted to persons who were destitute, and was obtainable only by order of a relieving officer; it also constituted the person admitted, or chargeable for the patient, a pauper, entailing the loss of privileges such as the Parliamentary vote. Many of the persons, however, for whom removal to hospital was desired on public-health grounds were above the pauper line, their removal being desired not because they had not proper lodging and accommodation at their own homes as far as their own welfare was concerned, but because they could not be treated at their homes without danger to others. The association of the fever wards with the workhouse, by position and administration, rendered their use distasteful to the independent artisan class and others of higher social position. Further, as it was found that there was a liability to the spread of infectious diseases, especially smallpox, to other inmates of the workhouse, even when the patients were treated in a separate building if the administration were in common with the rest of the establishment, it became the practice to exclude from the fever wards even pauper cases of infectious disease from the outside, reserving the wards solely for infectious cases originating in the workhouse.

For these reasons it often happened that there was no place to which a patient urgently requiring removal could be admitted, while at the same time there was at the workhouse infirmary accommodation for cases of infectious disease which had been provided at considerable cost lying idle and rarely used. The result outside London was to remove infectious cases from the workhouse, the sanitary authority providing hospitals to which infectious cases from all classes of the population might be sent. In London the investigations of the *Lancet* Commissioners, subsequently confirmed by a report to the Poor-Law Board by Dr. Edward Smith in 1866, on the condition of the Metropolitan workhouse infirmaries, led to the passing of the Metropolitan Poor-Law Act, 1867. This Act gave to the Poor-Law Board the power to combine the numerous Metropolitan parishes and unions previously maintaining their own poor into districts for the purpose of establishing asylums for the reception and relief of the sick and infirm, and under this power the whole of the unions and parishes in the Metropolitan area were combined by the Board into a single district for the purpose of providing hospitals for certain classes of sick and infirm poor persons chargeable to any union or parish in the district, among which classes were those who might be infected with or suffering from fever or smallpox. A board of managers was constituted consisting of representatives elected by the several unions and parishes, with a proportion nominated by the Poor-Law Board (now merged with the Local Government Board), and this body, the Metropolitan Asylums Board, thus formed purely for Poor-Law ends, has ultimately become the hospital authority for London for public-health purposes.

### Smallpox Epidemic of 1871-72.

The first steps taken by the Metropolitan Asylums Board were in view of an anticipated epidemic of relapsing fever in 1869, which, however, did not attain any serious proportions. But the great epidemic of smallpox in 1871-72 severely taxed the resources of the board, who hurriedly erected temporary structures, which speedily became crowded with patients. From that time till 1885 epidemics of smallpox recurred in London at intervals of about every three years, and typhus was also prevalent in the earlier years of the board's existence. The board had great difficulty in obtaining sites for hospitals, and some of their hospitals were closed wholly or in part, under legal injunctions, on the ground that they had caused excessive prevalence of smallpox in their neighbourhood. The number of patients for whom provision had to be made proved to be much larger than had been anticipated. It had been originally intended that the hospitals provided by the Asylums Board should be for paupers only, and that hospital accommodation for other classes of the community should be provided, as in other parts of the country, by the sanitary authorities, *i.e.*, the Metropolitan vestries. The vestries had the same power in this respect under the Sanitary Act, 1866, as sanitary authorities elsewhere, but very few of them made any such provision, partly on account of the difficulty of obtaining sites for hospitals within their districts, partly because by using the hospitals of the Asylums Board the expense of hospital provision could be thrown on the whole of the Metropolis. The attempt to limit admission to the Asylums Board's hospitals to destitute cases speedily broke down, and it was estimated that nine-tenths of the

* The previous article appeared on November 22.

persons admitted were not really paupers, but only became so on admission to the Board's hospitals.

The Commission of 1882.

A Royal Commission on Smallpox and Fever Hospitals which reported in 1882 recommended *inter alia* that the distinction between paupers and non-paupers should be abolished, and that there should be a single central authority for the purpose of providing hospital accommodation for all classes of the community. They recommended, moreover, that this authority should be the Metropolitan Asylums Board; but that it should be in its general aspect not a pauper but a sanitary authority, and that it should comprise representatives of the sanitary authorities as well as of the Guardians. This latter part of their recommendation has not been carried into effect. At the time that the Commission reported the London County Council did not exist, and the only representative body having jurisdiction over the whole Metropolis was the Metropolitan Board of Works, a body formed for a different purpose.

The restrictions upon the use of the Metropolitan Asylums Board's hospitals have, one by one, been removed since 1882. The Disease Prevention (London) Act, 1883, removed the civil disabilities which had till then been attached to admission into the Board's hospitals for infectious diseases. Subsequently the obtaining of an order of admission from the relieving officer was rendered no longer obligatory, and the certificate of any registered medical practitioner was accepted as evidence that the case was one suitable for admission. In 1888 the Board were authorised to admit diphtheria patients, and by the Poor Law Act, 1889, they were empowered to admit non-pauper cases of fever, diphtheria, and smallpox. The notification of infectious diseases was made compulsory in London in 1889.

By the Public Health (London) Act, 1891, the use of the Asylums Board's hospitals was in effect made free to all inhabitants of London reasonably believed to be suffering from fever, or smallpox, or diphtheria, the expense of maintenance being paid out of the Metropolitan common poor fund. In 1911 the Local Government Board, by Order, sanctioned the admission to the infectious hospitals of poor persons suffering from such infectious or contagious diseases, other than those above-mentioned, as they might thereafter determine, and in 1912 they authorised the admission of poor children suffering from measles and whooping-cough, and of poor persons suffering from puerperal fever, and also, subject to certain restrictions, of non-pauper cases of these diseases.

During the past thirty years there has been a large and progressive increase both in the total number of cases of infectious diseases admitted into the Asylums Board's hospitals and in the proportion of admissions to cases occurring. The proportion of admissions to the number of legally admissible cases notified was in 1890 33.6 per cent., and in 1912 84.8 per cent. The increase is due to the larger number of the infectious cases which now come to knowledge; to the more ample hospital accommodation now available; to the removal of restrictions and disqualifications, and of charges for admission; and to the greater appreciation by the public of the benefit of hospital treatment. The Board had in 1882 five hospitals for infectious diseases, containing in all 1,452 beds, exclusive of hospital ships. In 1912 they had thirteen hospitals for infectious diseases, with an aggregate of 8,206 beds; of these eight hospitals, with 3,847 beds, were for acute fever cases, two hospitals, with 2,269 beds, for convalescent patients, and three hospitals, with 2,090 beds, for smallpox. The accommodation is thus at the rate of one bed for every 550 inhabitants of London.

The earlier hospitals of the Board—and some of the later—which had to be hastily erected, under pressure of epidemics, were temporary wooden buildings; but these were found to be dangerous from the point of view of fire risk, expensive to maintain, and otherwise unsuitable; and the later fever hospitals have been well-designed structures of brick. Hospital ships were formerly used for smallpox cases, but the use of these has now been given up in favour of hospitals on shore, as more convenient.

The history of the Metropolitan Asylums Board's smallpox hospitals is noteworthy. So long as smallpox was treated in the Board's hospitals in London the disease continued more or less prevalent there year by year, with epidemic spread at intervals of about three years; and, as has been mentioned, there were complaints and litigation respecting the spread of smallpox around the hospitals. In 1882 the Royal Commission on Smallpox and Fever Hospitals recommended a limitation of the number of cases treated, that only the severe cases should be received into the town hospitals, the mild and convalescent cases being sent away to hospitals in the country or down the river. Smallpox nevertheless still continued prevalent until 1885, when the treatment of cases of smallpox in the town hospitals was given up, and all the patients were taken to floating hospitals down the river. This change was followed by an abrupt cessation of smallpox prevalence in London; and though minor epidemics, originating in imported infection, occurred in 1893-5 and in 1901-2, smallpox may now be said to be no longer an endemic disease in London.

The present practice is as follows: Upon the notification of a case of smallpox the patient is removed to a smallpox wharf. Here there are offices and a few beds in which patients can be detained for purposes of observation. The patient is examined by a medical officer, and if the diagnosis is confirmed—in a considerable number of cases it is found to have been mistaken—he is sent down the river in an ambulance steamer to the Asylums Board's pier at Long Reach near Dartford, about seventeen miles from London, whence he is conveyed to one or other of the Board's three smallpox hospitals in that neighbourhood.

The Asylums Board have country hospitals for convalescents from scarlet fever, diphtheria, etc. A system of motor-ambulances has recently been inaugurated.

